# A case of incubus phenomenon

**DOI:** 10.1192/j.eurpsy.2021.2162

**Published:** 2021-08-13

**Authors:** L. Tjokrodipo, A. Sneep, P. Michielsen

**Affiliations:** A-opleiding, GGZ Westelijk Noord-Brabant, Halsteren, Netherlands

**Keywords:** incubus phenomenon

## Abstract

**Introduction:**

The incubus phenomenon is a paroxysmal sleep-related disorder in which patients experience sleep paralysis and compound hallucinations. The symptoms of this phenomenon contain: sensed presence, fear, visual and auditory hallucinations, unusual body experiences such as out-of-body experience and feelings of floating/paralysis, experiencing a pressure on the thorax, difficulties breathing and a feeling of pain(1). This phenomenon appears to be universal, but has different cultural explanations(2).

**Objectives:**

We present a case of possible incubus phenomenon to raise awareness about this specific condition.

**Methods:**

A literature search in English was performed using PubMed with the following mesh term: ‘incubus phenomenon’.

**Results:**

We present a 29-year old man, known with an intellectual disability (IQ=74), psychotic disorder and a cannabis use disorder. After neurological examination, he was diagnosed with narcolepsy and cataplexy. Over the past weeks there had been an increase of hallucinations that appear before, during or after sleep. The patient’s thoughts included sexual approaches by caregivers, difficulties in breathing and a moving sensation while laying down in bed and experiencing pressure on the thorax assuming a woman was sitting on his chest. Literature search shows a lifetime prevalence of 0.11 % in general population versus 0.41 % in psychiatric patients(2.)

**Conclusions:**

Literature review shows only a few cases describing the incubus phenomenon. The prevalence is four times higher in patients with a psychiatric history(2) and should not be confused with psychotic disorder. Recognizing is important for proper treatment, as reoccurring attacks can be treated with anxiolytics, antidepressants, anticholinergics or anti-epileptics, and sleep hygiene methods(1.)

**Disclosure:**

No significant relationships.

